# Laparoscopic Cytoreduction Combined with Hyperthermic Intraperitoneal Chemotherapy (HIPEC) in Peritoneal Surface Malignancies (PSM): Italian PSM Oncoteam Evidence and Literature Review

**DOI:** 10.3390/cancers15010279

**Published:** 2022-12-31

**Authors:** Antonio Sommariva, Mario Valle, Roberta Gelmini, Marco Tonello, Fabio Carboni, Giovanni De Manzoni, Lorena Sorrentino, Enrico Maria Pasqual, Stefano Bacchetti, Cinzia Sassaroli, Andrea Di Giorgio, Massimo Framarini, Daniele Marrelli, Francesco Casella, Orietta Federici

**Affiliations:** 1 Advanced Surgical Oncology Unit, Unit of Surgical Oncology of the Esophagus and Digestive Tract, Veneto Institute of Oncology IOV-IRCCS, 35128 Padova, Italy; marco.tonello@iov.veneto.it; 2 Peritoneal Tumours Unit, IRCCS Regina Elena National Cancer Institute, 00144 Rome, Italy; mario.valle@ifo.it (M.V.); fabio.carboni@ifo.it (F.C.); orietta.federici@ifo.it (O.F.); 3 SC Chirurgia Generale d’Urgenza ed Oncologica, AOU Policlinico di Modena, 41125 Modena, Italy; roberta.gelmini@unimore.it (R.G.); lorena.sorrentino@live.it (L.S.); 4 Upper GI Surgery Division, University of Verona, 37134 Verona, Italy; giovanni.demanzoni@univr.it (G.D.M.); francesco.casella@univr.it (F.C.); 5 AOUD Center Advanced Surgical Oncology, DAME University of Udine, 33100 Udine, Italy; enricomaria.pasqual@uniud.it (E.M.P.); stefano.bacchetti@uniud.it (S.B.); 6 Colorectal Surgical Oncology, Abdominal Oncology Department, “Fondazione Giovanni Pascale” IRCCS, 80131 Naples, Italy; c.sassaroli@istitutotumori.na.it; 7 Surgical Unit of Peritoneum and Retroperitoneum, Fondazione Policlinico Universitario A. Gemelli—IRCCS, 00168 Rome, Italy; andrea.digiorgio@policlinicogemelli.it; 8 Surgery and Advanced Oncological Therapy Unit, Ospedale “GB.Morgagni-L.Pierantoni”—AUSL Forlì, 47121 Forlì, Italy; massimo.framarini@auslromagna.it; 9 Department of Medicine, Surgery, and Neurosciences, Unit of General Surgery and Surgical Oncology, University of Siena, 53100 Siena, Italy; daniele.marrelli@unisi.it

**Keywords:** peritoneal metastases, cytoreductive surgery, HIPEC, laparoscopy

## Abstract

**Simple Summary:**

Mini-invasive surgery represents an interesting yet challenging technical evolution for treating peritoneal metastases. This retrospective study aims to present the experience of the Italian Peritoneal Surface Malignancies Oncoteam with laparoscopic cytoreductive surgery (CRS) and hyperthermic intraperitoneal chemotherapy (HIPEC), including a detailed description of the technique and a systematic review of the literature. The study shows the feasibility and safety of laparoscopic CRS-HIPEC and its association with favorable outcomes in properly selected patients.

**Abstract:**

Cytoreductive surgery (CRS) and hyperthermic intraperitoneal chemotherapy (HIPEC) has gained increasing acceptance in clinical practice. Performing CRS and HIPEC laparoscopically represents a challenging and intriguing technical evolution. However, the experiences are limited, and the evidence is low. This retrospective analysis was performed on patients treated with laparoscopic CRS-HIPEC within the Italian Peritoneal Surface Malignancies Oncoteam. Clinical, perioperative, and follow-up data were extracted and collected on prospectively maintained databases. We added a systematic review according to the PRISMA method for English-language articles through April 2022 using the keywords laparoscopic, hyperthermic, HIPEC, and chemotherapy. From 2016 to 2022, fourteen patients were treated with Lap-CRS-HIPEC with curative intent within the Italian centers. No conversion to open was observed. The median duration of surgery was 487.5 min. The median Peritoneal Cancer Index (PCI) was 3, and complete cytoreduction was achieved in all patients. Two patients (14.3%) had major postoperative complications, one requiring reintervention. After a median follow-up of 16.9 months, eleven patients were alive without disease (78.6%), two patients developed recurrence (14.3%), and one patient died for unrelated causes (7.1%). The literature review confirmed these results. In conclusion, current evidence shows that Lap-CRS-HIPEC is feasible, safe, and associated with a favorable outcome in selected patients. An accurate patient selection will continue to be paramount in choosing this treatment.

## 1. Introduction

Peritoneal metastases (PM) represent a peculiar site of spread of a heterogeneous group of abdominal and extra-abdominal neoplasms [1]. Widely adopted cures are systemic chemotherapy and best supportive care, while the role of surgery is generally limited to diagnosis or palliative procedures. Cytoreduction (CRS) and Hyperthermic Intraperitoneal Chemotherapy (HIPEC) have gained increasing acceptance in clinical practice and have been adopted by most centers as the standard of care for pseudomyxoma peritonei (PMP) [2] and malignant peritoneal mesothelioma [3]. In ovarian cancer, several case-control studies and one randomized controlled trial showed that CRS-HIPEC gives a survival advantage in patients who underwent interval surgery [4,5], but also in the front-line approach and recurrent disease, the results are encouraging. The role of HIPEC in other PM, such as non-mucinous appendiceal, colorectal [6], gastric [7] sarcomatosis [8], and neuroendocrine [9], is still under study, and a clear benefit has been observed in selected cases. 

The technique of cytoreductive surgery for peritoneal surface malignancies (PSM) was standardized twenty years ago and consisted of up to six peritonectomy procedures in various combinations, visceral resection, and nodules “electroevaporation” on Glisson capsule and mesentery [10]. HIPEC is started immediately after completion of CRS and requires the insertion of intraperitoneal catheters percutaneously and is performed according to different drugs and perfusion schedules and conditions (temperature and duration). One of the most controversial issues of CRS-HIPEC is the significant morbidity profile related to the procedure. Recent evidence showed that grade III-IV morbidity and mortality rates after CRS-HIPEC are similar to those after other major abdominal surgical procedures [11].

Laparoscopy represents an important tool for diagnosing and staging PSM in patients selected for CRS-HIPEC [12]. Performing cytoreductive surgery and HIPEC laparoscopically represents a challenging and intriguing technical evolution, and the experiences are limited [13]. In the PSOGI registry, laparoscopic CRS-HIPEC (Lap-CRS-HIPEC), which is mainly tested in patients with a low peritoneal burden (PCI < 10) and low-grade histology, seems to reduce hospital stay through early recovery and low postoperative morbidity [14]. However, the evidence is still very limited, and several questions remain open, especially from an oncological point of view. Laparoscopic HIPEC without cytoreduction was tested as well for adjuvant or palliative purposes. In this setting, laparoscopy seems the ideal mean for HIPEC administration.

This study aims to evaluate indications, safety, and postoperative outcomes of patients treated with Lap-CRS-HIPEC in Italian peritoneal cancer centers. A systematic review is provided on the same topic.

## 2. Materials and Methods

### 2.1. Patients

A retrospective analysis of patients who underwent laparoscopic CRS and HIPEC in nine centers that are part of the Italian Peritoneal Surface Malignancies Oncoteam was performed. Eligibility for laparoscopic CRS-HIPEC was discussed at an interdisciplinary tumor board, considering clinical and pathological features and radiological findings. Laparoscopic CRS was performed according to the surgical principles described by Sugarbaker, which include resection of the greater and lesser omentum, the round ligament of the liver, and the adnexa in postmenopausal women, even if these organs are not involved by macroscopic disease [10]. We collected the following data: histopathology, age, gender, ECOG performance status, previous surgery, completeness of cytoreduction (CC), number of conversions to open surgery, PCI, neoadjuvant chemotherapy, number of peritonectomy procedures, number of resections, surgery time, intraoperative complications, perioperative blood transfusion, postoperative morbidity < 30 days (Clavien-Dindo) [15], recurrence, overall survival (OS) and disease-free survival (DFS). The study was approved by the Local Ethics Committee.

### 2.2. Literature Search

**Figure 1:**
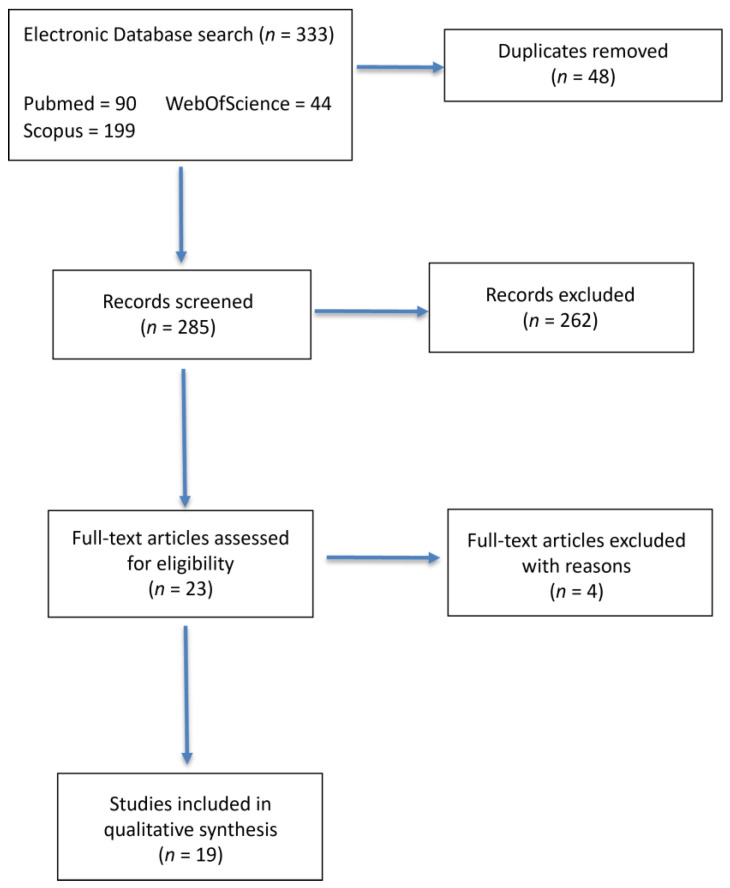
PRISMA 2009 flow diagram for literature search.

Searches in PubMed’s Medline (National Library of Medicine), Web of Science, and Scopus were performed for English-language articles through April 2022 using the keywords laparoscopic, hyperthermic, HIPEC, and chemotherapy (Figure 1). For each article, we collected the following data: name of the author, year of publication, type of study (with or without open control group), number of treated patients, and the same clinical data reported above. Exclusion criteria were the following: non-English papers, congress abstracts, editorials, comments, letters, review articles, or studies providing insufficient information were excluded. The systematic review followed the recommendations of the Preferred Reporting Items for Systematic Reviews and Meta-Analyses (PRISMA) [16]. The protocol has not been registered.

### 2.3. Statistical Analysis 

Statistical analysis was performed using IBM SPSS Statistics for Windows v.26.0 (IBM Corporation, Armonk, NY, USA). Continuous variables were reported as the median and interquartile range (25th percentile–75th percentile), while categorical variables were reported as frequency counts and percentages.

## 3. Results

### 3.1. Patients

From 2016 to 2022, fourteen patients were treated with laparoscopic CRS-HIPEC with curative intent. Clinical and perioperative data are reported in Table 1. Histological subtypes were heterogeneous and comprised five low-grade mucinous carcinoma peritonei (low-grade pseudomyxoma), one high-grade mucinous carcinoma peritonei (high-grade pseudomyxoma), two colorectal adenocarcinomas, one colorectal mucinous adenocarcinoma, two serous ovarian carcinomas, one neuroendocrine tumor, one goblet cells adenocarcinoma of the appendix and one appendiceal adenocarcinoma. Only three patients (21.4%) were administered neoadjuvant chemotherapy, one with colorectal cancer, one with ovarian cancer, and one with appendiceal adenocarcinoma. Nine patients (64.3%) underwent previous surgery with radical intent, two of which (14.3%) were treated with CRS and HIPEC (one was affected by pseudomyxoma, and one by serous ovarian carcinoma). 

### 3.2. Surgery

Laparoscopic cytoreductive surgery is a non-standardized surgical technique as it may vary according to the procedures needed to achieve complete cytoreduction. For this reason, the placement of trocars is modulated according to the main target. The patients were positioned in the gynecological position with appropriate devices to prevent slips from the operating table due to decubitus changes. Two video monitors were used. Pneumoperitoneum was established with the open technique in periumbilical, setting the intra-abdominal pressure at 14 mmHg. Under direct visualization, at least four other 10 mm balloon trocars were placed.

After a complete exploration of the abdominal cavity, any adhesions were sectioned, all abdominal compartments were explored, and the PCI was calculated.

**Figure 2:**
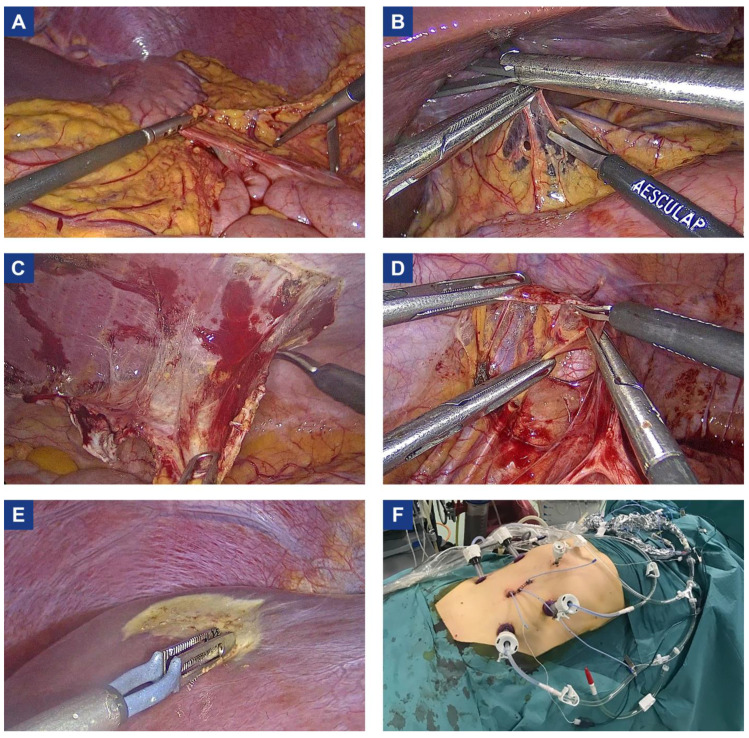
Representative images of laparoscopic cytoreductive surgery and HIPEC. (**A**) Greater omentectomy; (**B**) Lesser omentectomy; (**C**) Parietal peritonectomy; (**D**) Pelvic peritonectomy; (**E**) Glisson’s capsule electroevaporation; (**F**) HIPEC circuit.

Greater omentectomy was always performed. Gastroepiploic vessels (right gastroepiploic artery and vein) were sectioned at the origin (Figure 2A). The lesser omentum, the falciform ligament, and the round ligament of the liver were removed even in the absence of macroscopic disease (Figure 2B). Parietal peritonectomy (peritoneal stripping) was carried out as described by Sugarbaker [10]. Monopolar scissors were used to speed up dissection and simultaneously reduce blood loss through electrocoagulation (Figure 2C). The pneumoperitoneum makes the peritoneal stripping easier as it enlarges the virtual space between the peritoneum and posterior fascia or muscle. Under the diaphragm, dissection was carefully carried out to avoid the risk of perforation into the pleural space.

Pelvic peritonectomy was performed after identifying and preserving the structures of the retroperitoneum: ureter, common, external and internal iliac vessels and nerves (Figure 2D). The pelvic peritonectomy was completed by removing the pre-vesical and Douglas pouch peritoneum. At the end of the procedure, a leak test using saline solution and methylene blue was performed to exclude any bladder perforation. 

Single nodules in the intestine, mesentery, or liver surface can be easily electroevaporated (Figure 2E). Organ resections (e.g., colectomy, salpingo-oophorectomy) were performed with standard laparoscopic technique. Intestinal anastomoses were generally performed intra- or extra-corporeally through a mini median laparotomy (about 6–8 cm), according to the surgeon’s choice.

At the end of the cytoreductive phase, the circuit for HIPEC was settled in the standard fashion (Figure 2F). In the case of a mini-laparotomy, the skin was closed with a continuous suture to avoid any leakage. The catheters were placed inside the trocars. Optimal catheter placement was ensured by direct laparoscopic visualization. Temperature probes were inserted, usually one supra-mesocolic and one sub-mesocolic, in addition to those integrated into the circuit. HIPEC was performed using a perfusion machine supplied with a heater and a heat exchange connected to a dedicated system. Before starting HIPEC, the abdominal cavity was filled to assess the tightness of the system (no leakage from the trocars accesses and the laparotomic incision when present). After HIPEC, the abdominal cavity was laparoscopically explored to exclude thermal lesions. 

The median length of surgery was 487.5 min (IQR 433.8–567.5). Median PCI was 3 (IQR 2–4.5). Peritonectomy procedures were performed in eight patients (57.1%) and included seven pelvic peritonectomy, two right diaphragmatic peritonectomy, and five parietal peritonectomy. Visceral resections were performed in six patients (42.9%) and included three right hemicolectomies, one transverse colectomy, one ileocolic resection, and one colic wedge resection. Cytoreduction was complete in all the patients (CC = 0). Two intraoperative complications were described: one transverse colic ischemia requiring resection and one ileal and colon injury treated with sutures.

### 3.3. Postoperative Outcome

The median length of stay was six days (IQR 5–10.25). Two patients (14.3%) had grade III postoperative complications. One patient presented with enterorrhagia, which was treated endoscopically, and the other presented with jejunal perforation and fascial dehiscence and underwent reintervention.

### 3.4. Oncological Outcome

The median follow-up was 16.9 months (IQR 13.3–37.8). One patient died three months after surgery for unrelated causes (aspiration pneumonia). Eleven patients (78.6%) are alive without disease, whereas two patients (14.3%) developed recurrence: one patient with colorectal PM had lung progression, and one with ovarian PM had peritoneal and lymph node recurrence.

### 3.5. Review of the Literature

#### 3.5.1. Study Identification

The primary search identified 23 relevant publications based on information contained in the identified abstracts. Four studies were excluded after assessing the full-text version for the following reasons: three were about studies conducted on animals, and one was a systematic review. No other study was identified after reviewing the reference list. Therefore, 19 studies were included in the analysis [14,17,18,19,20,21,22,23,24,25,26,27,28,29,30,31,32,33,34]. Eight studies had a case-control design with open-CRS-HIPEC, four of which had a retrospective design [25,27,29,32], and the remaining were prospective studies [14,28,31,33]. No randomized clinical trials (RCT) were identified. The time of publication ranged between 2009 and 2020.

#### 3.5.2. Patients

The selected studies encompassed 482 patients (131 men, 291 women, and 60 not specified). The histopathology of the patients treated in the various studies includes ovarian, gastric, and colorectal PM, pseudomyxoma peritonei, and malignant mesothelioma (Table 2). The mean age was 53.35 years, and the mean BMI was 27.1. Only patients with an ASA score of 2 or lower were treated. All the patients underwent previous surgery before Lap-CRS-HIPEC. One hundred twenty patients were treated with neoadjuvant chemotherapy before the CRS and HIPEC.

#### 3.5.3. Intraoperative Data

The cytoreduction was complete in all the patients with no visible residual disease (Completeness of Cytoreduction, CC = 0) (Table 3). Only one patient in the open CRS-HIPEC arm of a comparative study underwent CC1 surgery [28]. The conversion rate to open-CRS-HIPEC ranged between 0 and 34.4%. In 15 studies (75%), the conversion rate was 0. The median PCI ranged between 1 and 5.56 in patients treated with Lap-CRS-HIPEC. In the comparative studies, the median PCI of open-CRS-HIPEC seems slightly higher (between 2 and 9.61) [14,25,27,28,29,31,32,33]. In the comparative studies, the surgery time ranged between 210 and 594 min for the laparoscopic approach and 240 and 438 min for the open approach. No intraoperative complications were described. Only two studies reported blood transfusion during the perioperative time [14,32]. Lap-CRS-HIPEC was performed using a median of 5 trocars placed at a variable quadrant of the abdominal wall in 13 studies [14,17,18,19,20,22,23,24,25,26,27,31,34]. Two studies reported using a single port [21,29], while one described a hand-assisted technique [33]. The extraction of the specimen was usually performed through a mini-laparotomy. Two studies reported natural orifice (vagina) specimen extraction [18,19]. Two hundred forty-seven visceral resections were described (113 omentectomies, 32 colectomies, 20 salpingo-oophorectomies, ten small bowel resections, seven recto-sigmoidectomies, four partial gastrectomies, 13 hysterosalpingo-oophorectomies). In nine studies, the administration of HIPEC was performed throughout the trocars [14,17,18,19,20,23,27,33,34]. All the other studies used the same trocar access but removed the devices before introducing the HIPEC catheters.

#### 3.5.4. Postoperative Outcomes

In the case-control studies, Clavien-Dindo grade III complications within three months from surgery were lower in patients treated with Lap-CRS-HIPEC (*n* = 7, 4.8%) compared to the open approach (*n* = 30, 13.5%). The complications described in patients treated with Lap-CRS-HIPEC were internal hernia, pleural effusions with the need for thoracic drainage, pelvic abscess with the need for percutaneous drainage [14,23], and a complicated hernia of port access [19]. In patients treated with the open technique, the complications reported were internal hernia, large bowel obstruction requiring sigmoidoscopic disimpaction, urinary obstruction requiring a percutaneous nephrostomy, bleeding requiring surgical reintervention, bowel leak, enterocutaneous fistulae from an ileum stump and colo-vesical fistulae [28,29,30,31]. There was no mortality within 30 days from surgery in all the studies. The hospital stay was shorter for Lap-CRS-HIPEC patients (range 3–12 days) than those treated with the open technique (range 4–19). Only four studies reported the postoperative time to chemotherapy. The mean time in weeks for Lap-CRS-HIPEC was 3.6, while for the open technique was 5.8 [19,24,29,32].

#### 3.5.5. Oncological Outcome

Ten studies reported the oncological outcomes in terms of months free from disease. According to Arjona-Sanchèz, at a mean follow-up of 32 months, 15 patients (16.5%) have developed recurrent disease [17]. Esquivel described six months free from disease after surgery in 2009 [22] and 17 months free from disease in 2012 [23]. Fagotti et al. reported ten months of follow-up with no secondary recurrences [24]. Gabriel, in 2019 described six months follow-up as negative for recurrence [26]. Mercier reported that one- and five-year OS are 100% in both laparoscopic and open groups. One- and five-year DFS are 100 and 91.04% in the laparoscopic group and 100 and 62.5% in the open group, respectively [28].

According to Passot, all patients were alive without recurrence, with a median follow-up of 192 days [31]. Rodriguez-Ortiz showed no significant differences in DFS, with 63.7% of the patients free from recurrence at 24 months in the open group and 71.4% in the Lap-CRS-HIPEC group. No deaths were registered in the laparoscopic group, while in the open group, 97.3% of the patients were alive 24 months after surgery [32]. Salti 2018 described 12 months free from recurrence, and Sommariva showed that all patients were free from disease after a median follow-up of 36 months. All patients showed negative tumor markers and no evidence of recurrence on the CT scan [33,34].

## 4. Discussion

Laparoscopic surgery is nowadays the standard treatment in most abdominal tumors with the same oncologic outcomes as the open technique but with the great advantage of faster postoperative recovery and shorter hospital stay. In highly selected patients, the laparoscopic technique can lead to positive oncological outcomes even for CRS and HIPEC [32]. The postoperative recovery after mini-invasive surgery is faster, with lower pain and incidence of postoperative ileus, a quicker return to solid feeding and systemic chemotherapy, and a significant decrease in postoperative complications [35,36]. 

Our clinical experience and the literature review confirmed that the lap CRS-HIPEC is feasible and reliable for treating selected patients with PSM. Surgical time and intraoperative complications are very limited. Moreover, the conversion rate is very low and lower than 10% in most clinical series. The postoperative clinical course is safe with a very low rate of severe complications. In the few comparative studies, the length of stay is lower than in the open approach. Indeed, the faster return to systemic chemotherapy after laparoscopic cytoreduction and HIPEC is an essential advantage of the mini-invasive approach over the open technique [32,37]. 

The past concerns for survival rates, port site recurrence, and regional or distant recurrence, have been proved wrong, with no substantial differences between laparoscopic and open techniques [38,39]. However, this aspect should be better defined in studies with longer follow-ups. Conversely, Lap CRS-HIPEC could have a potential oncological advantage as some experimental and clinical data showed that intraabdominal hyper-pressure could potentially increase drug penetration, better heat preservation, and a more homogeneous drug distribution over the peritoneal surfaces [40,41]. The pharmacokinetics of drugs during laparoscopic HIPEC should be compared to the open technique in further studies in terms of peritoneal exposure to the drug and systemic absorption. In the future, further indications of HIPEC could be proposed. Lap-HIPEC has been applied as a palliative treatment of hemorrhagic and refractory ascites with encouraging results [9,18,25,42,43]. Moreover, recent studies showed the effectiveness of Lap-HIPEC as a neoadjuvant treatment, converting peritoneal cytology (from positive to negative) and reducing PCI [44]. 

CRS-HIPEC needs standardization from evidence and expert consensus [45]. For laparoscopic CRS-HIPEC, this need is relatively more stringent, not only for the technique but also for the indications.

Peritoneal Cancer Index (PCI) and histology are the main selection criteria for Lap-CRS-HIPEC. The initial applications in low-grade tumors such as pseudomyxoma or benign multicystic mesothelioma have been extended to peritoneal metastases originating from ovarian, gastric, and colorectal cancer with favorable oncological results [23,24,25,26,27,28,29,30,31], showing that the tumor histology should not be considered an absolute contraindication to laparoscopic CRS-HIPEC. Regarding PCI, in all comparative studies (Lap versus Open-CRS-HIPEC), there is a large difference between the two groups. The Peritoneal Cancer Index (PCI) of patients treated with Lap-CRS-HIPEC never exceeds 10, showing that this cut-off value for Lap-CRS-HIPEC is a solid selection criterium for guaranteed low conversion and complications rate with an optimal oncological result. 

Our study has several limitations. Most of the studies found in the literature are retrospective studies with a small sample size. Further, multi-institutional studies and randomized controlled trials are needed to evaluate the effectiveness of Lap-HIPEC and its various applications. An accurate patient selection will continue to be paramount in choosing this treatment.

## 5. Conclusions

Current evidence shows that Lap-CRS-HIPEC is feasible, safe, and associated with favorable postoperative outcomes in selected patients affected by PSM. Oncological outcomes of patients treated with Lap-CRS+HIPEC have yet to be established, and the procedure can be proposed in patients with low biological aggressiveness (pseudomyxoma, multicystic mesothelioma) and low PCI.

## Figures and Tables

**Table 1:** Clinical data of patients treated with Lap-CRS-HIPEC.

Patients Characteristics (*n* = 14)	
Age, years (median, IQR)	61.0 (51.75–73.50)
Gender (*n*,%)	
Male Female	5 (35.7) 9 (64.3)
Primary tumor (*n*,%)	
Pseudomyxoma Colorectal Ovarian NET Appendix	6 (42.9) 3 (21.4) 2 (14.3) 1 (7.1) 2 (14.3)
ECOG 0 (*n*,%)	14 (100.0)
Neoadjuvant chemotherapy (*n*,%)	3 (21.4)
Status of PM	
Synchronous Metachronous	0 (0.0) 16 (100.0)
Conversion to open (*n*,%)	-
CC 0 (*n*,%)	14 (100.0)
PCI (median, IQR)	3 (2–4.25)
Surgery time, minutes (median, IQR)	487.5 (433.8–567.5)
Peritonectomy Procedures (*n*,%)	8 (57.1)
Visceral Resections (*n*,%)	6 (42.9)
Blood transfusion (*n*,%)	2 (14.3)
Length of stay, days (median, IQR)	6 (5–10.25)
Minor complications, grade I–II (*n*,%)	2 (14.3)
Major complications, grade III–IV (*n*,%)	2 (14.3)
90-days mortality (*n*,%)	-

**Table 2:** Preoperative data.

Author, Year	Patients	Histopathology	Previous Surgery	Neoadjuvant Chemotherapy
	Lap/Open		Lap/Open	Lap/Open
	*n*		*n*	*n*
Abudeeb, 2020 [14]	55/29	LAMN-II	55/29	0/0
Arjona-Sánchez, 2019 [17]	90	Multiple histologies	81	NA
Arjona-Sánchez, 2019 [18]	6	Ovarian	NA	6
Arjona-Sánchez, 2020 [19]	12	Multiple histologies	NA	6
Bãlescu, 2017 [20]	2	Gastric	2	2
Dumont, 2020 [21]	12	Pseudomyxoma, mesothelioma	2	0
Esquivel, 2009 [22]	1	Mesothelioma	1	NA
Esquivel, 2012 [23]	19	Pseudomyxoma	19	0
Fagotti, 2014 [24]	10	Ovarian	10	10
Fagotti, 2015 [25]	11/11	Ovarian	11/11	11/11
Gabriel, 2019 [26]	1	Pseudomyxoma	1	0
Koti, 2020 [27]	7/38	Multiple histologies	NA	NA
Mercier, 2020 [28]	32/11	Pseudomyxoma, mesothelioma, ovarian	NA	0
Morton, 2021 [29]	10/40	Ovarian	NA	10/40
Parkin, 2019 [30]	1	LAMN-II	NA	NA
Passot, 2014 [31]	8/41	Pseudomyxoma, mesothelioma	49	NA
Rodríguez-Ortiz, 2020 [32]	18/42	Multiple histologies	12/24	8/30
Salti, 2018 [33]	11/11	Pseudomyxoma, colorectal	NA	3/3
Sommariva, 2019 [34]	3	LAMN-II	3	0

Abbreviations: LAMN, low-grade appendiceal mucinous neoplasm.

**Table 3:** Intraoperative and postoperative data.

Author, Year	Patients	CC 0	Peritoneal Cancer Index	Surgery Duration	Morbidity (Grade ≥ III) * within 90 Days from Surgery	Mortality within 30 Days from Surgery	Length of Hospital Stay	Time to Chemotherapy
	Lap/Open	Lap/Open	Lap/Open	Lap/Open	Lap/Open	Lap/Open	Lap/Open	Lap/Open
	*n*	*n*	Median PCI	Minutes	*n*	*n*	Days	Weeks
Abudeeb, 2020 [14]	55/29	55/29	1/2	528/438	5/9	0/0	6/10	NA
Arjona-Sánchez, 2019 [17]	90	90	4.1	282	0/9	0	7.4	NA
Arjona-Sánchez, 2019 [18]	6	6	<10	480	0	0	5	NA
Arjona-Sánchez, 2020 [19]	12	12	4.5	468	2	0	5.5	3.5
Bãlescu, 2017 [20]	2	2	4.5	336	0	0	NA	NA
Dumont, 2020 [21]	12	12	2	240	2	0	8.5	NA
Esquivel, 2009 [22]	1	1	4	NA	0	0	5	NA
Esquivel, 2012 [23]	19	19	4.2	270	1	0	5.3	NA
Fagotti, 2014 [24]	10	10	2	180	0	0	4	3
Fagotti, 2015 [25]	11/11	11/11	2/2	180	0	0	4/8.5	NA
Gabriel, 2019 [26]	1	1	1	426	0	0	4	NA
Koti, 2020 [27]	7/38	7/38	5.56/9.61	594/468	0/3	0	NA	NA
Mercier, 2020 [28]	32/11	32/10	2.5/7	210/240	0/1	0	11/13	NA
Morton, 2021 [29]	10/40	10/40	NA	324/336	0/4	0	3/4	26/30
Parkin, 2019 [30]	1	0	5	510	0	0	5	NA
Passot, 2014 [31]	8/41	8	2.5	210/240	1/4	0	12/19	NA
Rodríguez-Ortiz, 2020 [32]	18/42	18/42	3/5	420/306	1/9	0/0	4.5/8	4/7
Salti, 2018 [33]	11/11	11/11	4.1	312/288	0/0	0/0	6.5/9.1	NA
Sommariva, 2019 [34]	3	3	1	NA	0	0	10	NA

Abbreviations: CC, completeness of cytoreduction. * According to the Clavien-Dindo classification.

## Data Availability

The data presented in this study are available on request from the corresponding author.
